# Early Cell Death Detection with Digital Holographic Microscopy

**DOI:** 10.1371/journal.pone.0030912

**Published:** 2012-01-31

**Authors:** Nicolas Pavillon, Jonas Kühn, Corinne Moratal, Pascal Jourdain, Christian Depeursinge, Pierre J. Magistretti, Pierre Marquet

**Affiliations:** 1 Microvision and Microdiagnostics Group (MVD), STI, Ecole Polytechnique Fédérale de Lausanne (EPFL), Lausanne, Switzerland; 2 Département de Psychiatrie-CHUV, Centre des Neurosciences Psychiatriques, Site de Cery, Prilly, Switzerland; 3 Brain Mind Institute, Ecole Polytechnique Fédérale de Lausanne (EPFL), Lausanne, Switzerland; City of Hope National Medical Center and Beckman Research Institute, United States of America

## Abstract

**Background:**

Digital holography provides a non-invasive measurement of the quantitative phase shifts induced by cells in culture, which can be related to cell volume changes. It has been shown previously that regulation of cell volume, in particular as it relates to ionic homeostasis, is crucially involved in the activation/inactivation of the cell death processes. We thus present here an application of digital holographic microscopy (DHM) dedicated to early and label-free detection of cell death.

**Methods and Findings:**

We provide quantitative measurements of phase signal obtained on mouse cortical neurons, and caused by early neuronal cell volume regulation triggered by excitotoxic concentrations of L-glutamate. We show that the efficiency of this early regulation of cell volume detected by DHM, is correlated with the occurrence of subsequent neuronal death assessed with the widely accepted trypan blue method for detection of cell viability.

**Conclusions:**

The determination of the phase signal by DHM provides a simple and rapid optical method for the early detection of cell death.

## Introduction

Cell death can be caused by activation of distinct molecular pathways, including apoptosis, necrosis, and autophagy which are characterized by a distinct set of temporal, morphological, biochemical, and gene expression features [1], [2]. Although, in a whole organism, certain types of cell death such as apoptosis result in the controlled breakdown of the cell avoiding any intracellular medium release, *in vitro*, the different cell death pathways proceed to an end-stage called secondary necrosis, which shares many features with primary necrosis pathway, in particular the loss of cell membrane integrity and the subsequent release of the cellular content into the surrounding extracellular space [1], [3]. Consequently, *in vitro*, assays usually differentiate between viable and non-viable cells by assessing membrane integrity thanks to inclusion and/or exclusion dyes such as trypan blue or propidium iodide [4], [5], or the detection of specific intracellular compounds in the surrounding medium (lactate deshydrogenase release) [6]. However, depending on the stimulus that induced cell death, such cell viability assays assess a late stage of the cell death processes with extrinsic contrast agents and usually require several steps (washing, harvesting, solubilization, etc.) which take several hours for completion [7].

Specific morphological features, in particular volume changes accompany cell death processes and are often used to define the different cell death pathways [1]. For example, the loss of cell volume or cell shrinkage that occurs during apoptosis is a key morphological characteristic separating this physiological cell death process from necrosis, characterized by an initial cell swelling. However, minor variations in cell volume occur physiologically; thus cells have volume regulatory mechanisms to compensate for these physiological variations in order to maintain an appropriate balance of ions across their cell membrane [8]. Within this framework, it has been recently stressed that it is less the cell volume variations (shrinkage, swelling) which play a critical role in the activation/inactivation of the cell death processes than the cell's capability to successfully regulate its volume through the activation of various ionic pathways in order to return to a near normal size and above all to maintain ionic homeostasis [9], [10]. Practically, if volume regulatory mechanisms are inactivated or overridden, resulting in an uncontrollable change in volume with an alteration of ionic homeostasis, cell death processes are activated.

In this article, we present a methodology to rapidly assess cell viability *in vitro* based on the ability of digital holographic microscopy (DHM) to quantitatively and dynamically measure cellular shape and volume with a high sensitivity [11]–[14]. Thanks to an imaging approach based on digital procedures, DHM presents various advantages for automatic optimization of imaging conditions [15], [16], and the development of dedicated automated detection methods [17]–[19], and enabled measurement and characterization on various types of cells such as red blood cells [20], myoblasts [21] or sperm cells [22], for instance.

Practically, DHM is used to monitor early cell volume regulation (CVR) processes in response to specific events likely to induce cell death. The efficacy of these volume regulatory processes is correlated with the occurrence or not of a subsequent cell death assessed with a standard trypan blue staining test.

Concretely, we have examined the glutamate-mediated excitotoxicity, a well-established form of neuronal death involved in neurodegenerative and ischemic conditions of the central nervous system (CNS), which trigger apoptosis or necrosis pathways [23], [24]. Glutamate-mediated excitotoxicity is characterized in particular by a neuronal swelling resulting from transmembrane water movements accompanying a strong increase of the Ca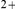 and Na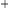 intracellular concentrations [25]. In the experiments presented in this article, the neuronal CVR has been systematically studied, using the high sensitivity of the DHM quantitative phase signal [11] and correlated with the subsequent occurrence or not of a delayed neuronal death.

## Results

### Phase signal measurement

In the different experiments described below, the primary signal of interest is the time-course of the mean phase shift value measured on cell bodies. Practically, the phase shift induced on the transmitted wave arises from the difference in refractive index (RI) between the specimen and the surrounding medium and is proportional to the thickness of the observed transparent specimen [12]. The phase value 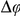 can thus be expressed as
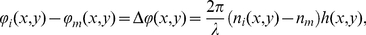
(1)where 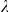 is the wavelength of the illumination light, 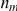 is the RI of the perfusion solution, 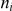 is the mean intracellular RI along the optical path length and 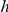 is the thickness of the cell at position 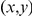 in the field of view. Throughout this article, the phase shift corresponds to a spatial averaging over a constant surface localized within the cell body and is referred to as the phase signal.

As the experiments are performed during several hours, the preparation can move during time, because of mechanical relaxation due for example to temperature changes or cellular movements. The position variations in the 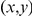 plane perpendicular to the light propagation direction are compensated simply by tracking the cells during measurements. Furthermore, the movements along the 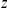 axis can be digitally compensated thanks to the digital focusing capabilities of DHM [26]. Mechanical refocusing was only performed during assessment of cell viability, in order to ensure focused images for bright field color acquisitions.

### Cell viability and control

Control experiments were performed to assess effects of the reagent on the phase signal and the lack of toxicity of the trypan blue protocol used, since cytotoxic effects following long exposures to the reagent have been described [27], [28]. Practically, cells were periodically immersed in the reagent medium during 3 minute periods and the DHM phase signal measured simultaneously with trypan blue reactivity.

**Figure 1:**
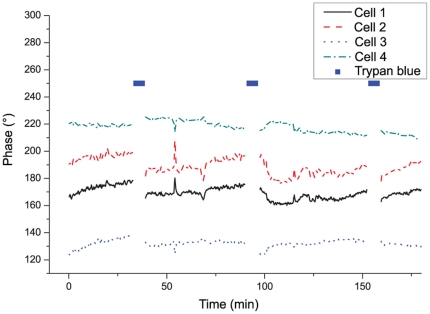
Control experiment. Control experiment to test for possible effects on the phase signal by periodic reagent immersion, measured on several cell bodies.

The typical phase signal measured on four cell bodies is shown in Fig. 1 (curves representative of approximately 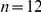 cells), while reagent has been periodically applied for 3 minute periods separated by one hour interval. One can identify a small phase signal change after the application of the reagent (typically at approximately 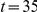 and 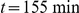), which corresponds to the consequence of a small osmotic shock, as the osmolarity of the perfusion medium is slightly changed by the 1∶10 dilution of reagent. These variations are however within the range of typical phase fluctuations exhibited by “healthy” living neurons in culture, and are gradually regulated as it can be seen by the slow recovery of the phase signal after each application.

**Figure 2:**
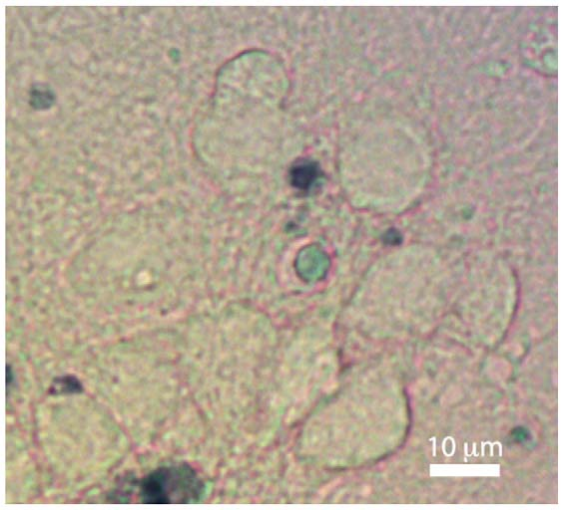
Color image of cells of control experiment. Color image of the cells monitored in Fig. 1 at 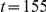 min, where cells are viable and not stained after repeated exposure to the exclusion dye.

This experiment shows that repeated exposure to the dye does not lead to a modification of cell morphology nor to a significant cell staining even several hours after the first application, as shown in Fig. 2, where cells were exposed to three trypan blue applications over a 155 minute interval are presented. The stained biological material corresponds to small fragments originating from cells already dead before the beginning of the experiments. This stresses that within the typical time-range of the experiments presented in this article, repeated trypan blue applications of 3 minutes do not lead to a significant cellular staining.

### Excitotoxicity measurements

We measured the effect of glutamate application to cell cultures in several conditions, notably by changing the application duration (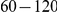 seconds) and concentration (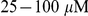).

**Figure 3:**
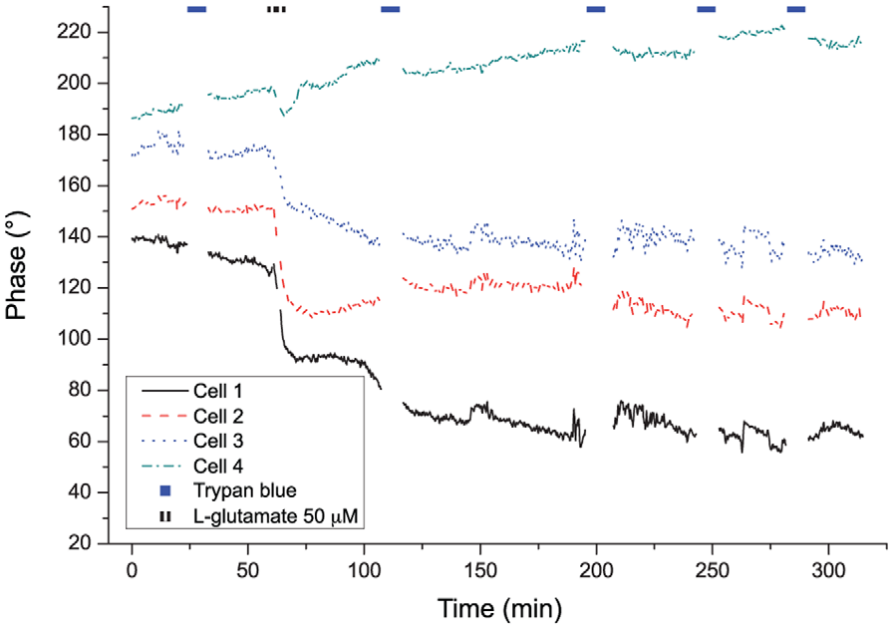
Cellular phase signal. Monitoring over time of the phase signal on several cells under a (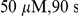) glutamate pulse.

**Figure 4:**
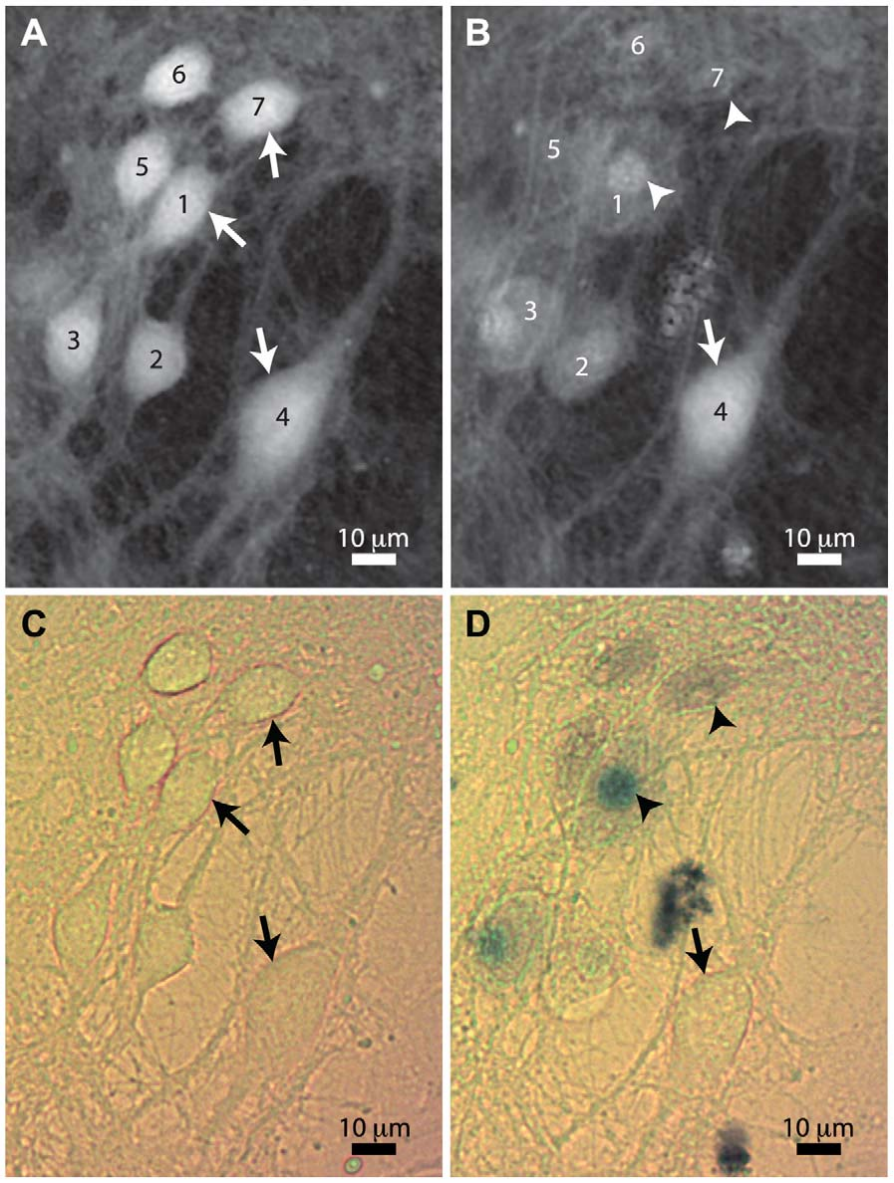
Images corresponding to experiment of **Fig. 3**. Cells corresponding to the experiment depicted in Fig. 3 when the glutamate pulse occurred at 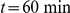. Phase images at **A** 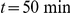 (before stimulation), and **B** at 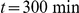 (after stimulation); note that cells 1–3 present morphological changes such as round cell shape or nucleus condensation, while cell 4 appears normal. Color images at **C** 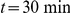 and **D** 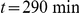: note cells 1–3 clearly stained by trypan blue, unlike cell 4. Some viable and dead cells are shown by respectively arrows and arrowheads in all subfigures.

We present in Fig. 3 an experiment where a glutamate pulse (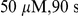) was applied to the culture. The phase signals presented correspond to the cells shown in Fig. 4A. Among the four curves shown, three (cells 1, 2, 3) present a strong and irreversible phase signal drop comprised between 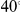 and 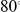, while the phase signal of cell 4 recovers, after a small transient decrease (around 10 degrees), over a period of time of approximately 10 minutes.

Bright field color images of the culture are presented in Figs. 4C–D, respectively at 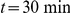 before the glutamate application and 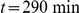, when non viable cells are identified by the blue staining. The study of the images of Fig. 4 shows that cells 1–3 are stained and present strong morphological changes after glutamate application, while cell 4 remains unstained with a stable unchanged morphology.

In Fig. 4, one can identify at the top of the field of view cells which exhibit a faint staining of exclusion dye (cf. Fig. 4D, cells 5–7) while not producing any significant phase signal in Fig. 4B at 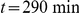. These three cells produced an irreversible phase drop after glutamate exposure, followed by a sudden second drop leading to their near disappearance in phase images. These cells were stained very rapidly after glutamate exposure, and lost all cellular morphological characteristics at this time.

**Figure 5:**
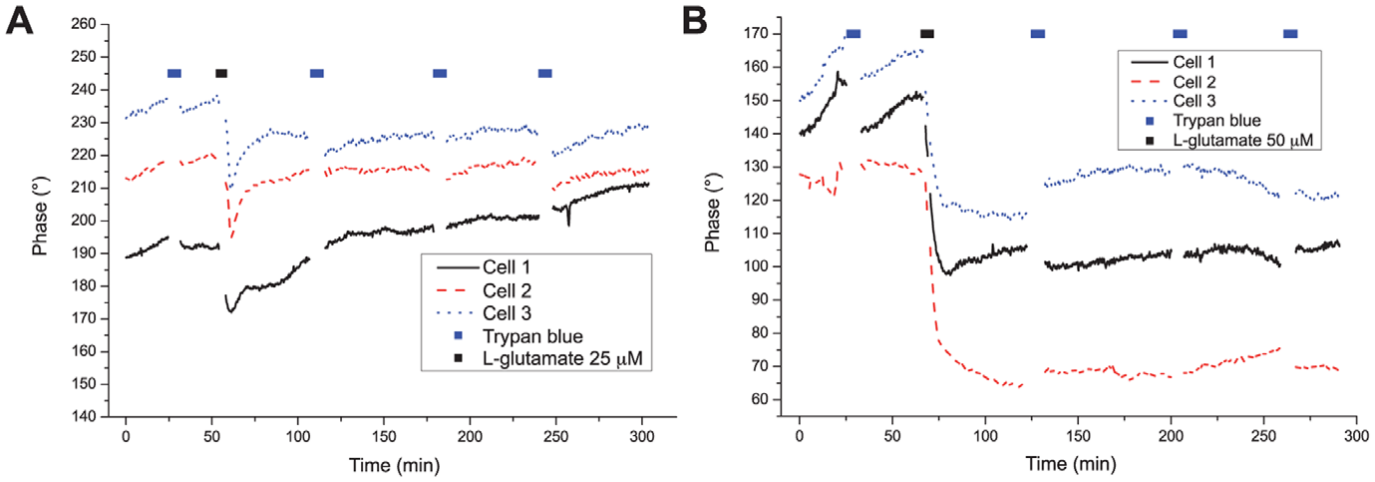
Phase responses resulting from different glutamate applications. **A** reversible responses (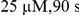 glutamate pulse), and **B** irreversible responses (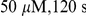 glutamate pulse). Both types of phase curves are representative of measurements on 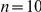 cells each.

The results presented in Fig. 3 could be reproduced on several cultures with concentrations ranging from 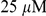 to 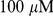 (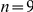 for a total of 71 cells), as shown for typical responses in Fig. 5, where slightly different parameters of stimulation were employed. Thus, a glutamate pulse of 90 seconds (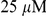) produced reversible phase responses, similar to the one of cell 4 in the previous experiment (cf. Fig. 5A). The viability of cells could be confirmed by trypan blue exclusion up to 4 hours and 30 minutes after stimulation, a time at which the experiment was interrupted, with all cells being unstained and having a morphology similar to the one before the onset of the glutamate perfusion (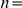
6). In Fig. 5B, a pulse of 120 seconds (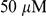) produced irreversible phase drops of typically 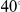 to 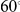. Cell death could be confirmed by a positive staining 3 to 4 hours after the stimulation (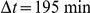 for cells 1 and 2, and 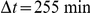 for cell 3). These results are representative of 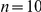 cells in this culture.

Thus two main types of signals can be seen after glutamate application to neurons: either a large irreversible phase drop or a reversible response characterized by a phase recovery. These two types of signals, observed within 10 to 20 minutes after the glutamate application onset, are in very good agreement with viability assessment performed with trypan blue staining several hours later. Indeed, cells expressing an irreversible phase drop are later stained, while those exhibiting a phase recovery remain unstained up to the end of the experiment. In both cases, the two different phase responses to glutamate application stabilize to a steady-state phase value after 10–20 minutes, either to a value similar to the one before application (reversible) or smaller by tens of degrees (irreversible), enabling discrimination between the two responses within a maximum of 20 minutes. This temporal behavior is also reproduced in experiments with various concentrations and application durations (cf. Fig. 5A–B).

In another set of experiments, we aimed at determining how fast trypan blue stains non-viable cells, in order to ensure that no staining was missed because of the interruption of the experiment. Various studies stressed that the glutamate concentration influences mainly the amount of dead cells after exposure, rather than the time required to obtain trypan blue staining. Practically, prolonged glutamate applications (30 min) with various concentrations (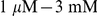) were typically not inducing significant new staining of trypan blue 3 hours after application [23]. The fact that most glutamate-induced cell deaths can be detected within the first 3 hours was also corroborated at 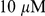 for lower application durations such as 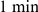 [29].

**Figure 6:**
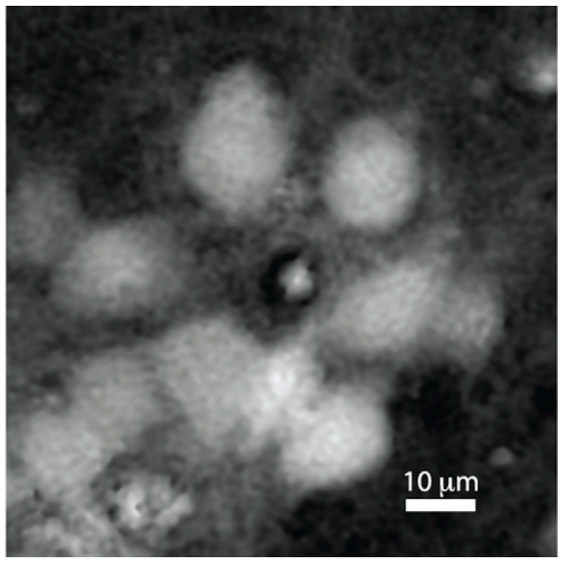
Quantitative phase image of cells. Quantitative phase image of the cells corresponding to the control experiment (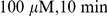), ensuring no missed staining during a typical measurement duration. The figure corresponds to Video S1 containing the evolution in time of neuronal body shapes during control and excitotoxic stimulation.

Accordingly, an experiment performed with a glutamate application (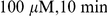), following by repeated dye immersions every hour, showed that over 90% of cells were stained by trypan blue after 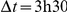. The phase image corresponding to a part of the monitored field of view is presented in Fig. 6, and the embedded media file makes possible to identify the morphological changes ongoing after the glutamate application.

## Discussion

As expressed in Eq. (1), the phase signal is dependent on both the thickness and RI of the cells, related to the intracellular content. Both parameters are indicators of the regulation capability of cells, as volume changes are essentially driven by water movements induced by ionic homeostasis mechanisms. These mechanisms also influence the RI through dilution of the intracellular content, particularly proteins. This implies that the phase signal is influenced by both parameters during a glutamatergic stress, which is characterized by a cell swelling and an entry of water, leading to an intracellular content dilution. We have previously shown that phase signal changes mainly reflect intracellular RI variations, due to the closeness of the refractive index values 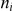 and 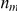 [11], in agreement with other studies [30]. Accordingly, decoupling procedures confirmed that the large phase decrease monitored during the well-known glutamate-mediated neuronal volume swelling, induced by a water inflow accompanying 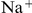 and 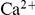 flux [25], results from an intracellular RI decrease [11], [14], [31].

Thus it clearly appears that the phase signal is mainly sensitive to any process that modifies the intracellular protein concentrations, including transmembrane water movements, which accompany various ionic fluxes involved in the maintenance of ionic homeostasis. The phase signal can thus provide information on both cell morphology as well as homeostasis mechanisms coupled to CVR. Furthermore, it has also been shown that inefficient CVR is very likely to lead to a subsequent cell death [10]. Consequently DHM, by allowing an accurate monitoring of CVR, is used here to derive a criterion for early cell death detection.

In the experiments presented in the excitotoxicity section, two kinds of glutamate-mediated phase signals are revealed: a large and irreversible phase drop and a reversible response corresponding to a phase recovery. The latter, also characterized by a rapid neuronal morphology recovery, is compatible with an efficient ionic homeostasis. In contrast, the irreversible phase drop can be interpreted as a persistent dysregulation of the intracellular ionic homeostatic state underlying the observed irreversible cell volume swelling. Furthermore, some cells encounter after the irreversible phase drop a dramatic loss of phase signal (cf. cells 5–7 in Fig. 4), which is very likely due to a large dilution of the intracellular RI, consistent with the loss of cellular morphology observed in Fig. 4D. This rapid dilution can be interpreted as a lysis following a rapidly-triggered excitotoxicity [25]. Consistent with suggestions made by Hoffmann et al. and Chen et al. [9], [10], we consider the irreversible phase drop as an early indicator of subsequent cell death mechanisms triggered by a persistent ionic homeostasis dysregulation.

Practically, the discrimination between the two signal types can be easily achieved. Indeed, the magnitudes of the two phase responses are dramatically different (several degrees for reversible responses, several tens of degrees for the irreversible ones), and their morphology are extremely dissimilar.

These considerations allow to define simple criteria that can be applied to automated early cell death detection by DHM. For instance, the difference in quantitative phase values before and after glutamate application, the aspect of the temporal phase signal, being either a bell-shaped curve in the case of a reversible response, or a strong phase drop in the other, can be easily implemented as an automatic detection tool. Typically, a threshold value can be defined at 20 degrees, clearly separating the reversible responses in the 10 degrees range, and irreversible responses of 40–80 degrees, leading to an automated detection which could yield applications for high throughput screening of bioactive compounds. This measurement approach could indeed be extended to several fields of view or multiple wells measurements through automation, where DHM measurement could bring various advantages in this context, such as digital focusing, which enables the retrieval of in-focus images at post-processing level, reducing the mechanical constraints during measurements [32], or label-free detection.

Overall, the data presented in this article indicate a remarkable concordance between assessment of cell viability by dye inclusion and phase signal monitoring by DHM for cell death detection. Cells identified by the phase signal as not efficiently regulating their volume are detected as non-viable cells several hours later through trypan blue staining. Experiments show that the different phase responses stabilize to steady states values within tens of minutes, thus defining the rapidity of detection of DHM, in contrast to dye assessment which requires typically several hours before identification of cell death.

This strong difference in detection time can indeed be explained by the very different principles on which both methods rely. The phase signal is highly sensitive to CVR processes related in particular to water transmembrane movements associated with ionic homeostasis, a condition necessary to prevent the activation of cell death processes [9], [10]. In contrast, trypan blue relies on the loss of membrane integrity which occurs as a consequence of necrosis, occurring either as the primary cause of cell death, or as a secondary process *in vitro* [1], resulting in nuclear staining. The loss of membrane integrity corresponds to a late stage of the cell death process, occurring with a delay of several hours (typically 1 to 3 hours) after exposure to strong glutamatergic stimulation (e.g. 100 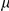
M–3 mM, 30 min), known to induce necrosis or apoptotic deaths in cortical culture [23]. One should note also that inclusion dyes such as trypan blue or propidium iodide require a long time in order to stain the nucleus, as the dye must pass through both the cell and nuclear membranes.

As far as glutamate-mediated excitotoxicity is concerned, cell death occurs both through necrosis and apoptosis with different time-courses, depending on glutamate concentration. Glutamate exposure leading to cell death triggers calcium uptake in mitochondria [33], resulting in ATP depletion [34].

In the experiments presented in this article, several indicators seem to suggest that apoptotic mechanisms are at least partly involved in the cell death process. Apoptosis is indeed characterized mainly by cell shrinkage, nuclear condensation, and cell fragmentation. The nucleus condensation can be readily identified in most of the observed cells, by considering the phase shift induced by the nucleus in phase images (see cells 1–3 in Fig. 4B). Another indicator of apoptotic behavior consists in the formation of blebs which are gradually retracted after glutamate application, as can be observed in the embedded file of Fig. 6. These characteristics, which were identified in a vast majority of the observed cells in the various experiments presented, are to be compared with the absence of visible nucleus and the diffuse blue staining of cells 5–7 at the top of the field of view presented in Fig. 4. These cells may have been the site of a purely necrotic death followed by a lysis, as no nucleus condensation can be identified, even though staining occurred, and their intracellular content suddenly disappeared in phase imaging.

Other typical indicators of apoptotic cell death were not identified in our experiments, such as cell shrinkage or fragmentation. The ATP depletion induced by mitochondrial membrane depolarization could impede the continuation of apoptotic mechanisms, preventing cell fragmentation, and thus finally leading to necrosis [34]. Finally, one should note finally that cells considered as viable by the absence of trypan blue staining and efficient CVR processes do not present any morphological indicators of cell death triggering, neither necrotic nor apoptotic. Typically, no nucleus condensation is observed, and the cell body does not present the typical spherical shape of necrotic death.

Thus this article describes a novel optical method for the early detection of cell death. In addition, even though the goal of this study was not to discriminate between the different cell death pathways, a finer analysis of the early phase signal could potentially be conveniently used for the detection of various cell death types. As purely necrotic death is characterized by the loss of homeostasis capability and subsequent swelling followed by lysis, while purely apoptotic death can be distinguished by a dramatic cell shrinkage, a method based on the detection of CVR may enable to differentiate, at an early stage, the two different cell death forms through the quantitative measurements of specific morphological features. Other studies, studying specifically an apoptotic behavior on oligodendrocytes by employing staurosporine, indeed showed an increase of the phase signal, consistent with a cell shrinkage and a consequent increase in intracellular RI [30].

### Conclusion

We presented in this article a method allowing the early detection of cell death through a quantitative phase measurement by digital holographic microscopy (DHM). Cell death was triggered in primary cultures of mouse cortical neurons by applying excitotoxic concentrations of glutamate. Practically, cell volume regulation (CVR) was monitored by DHM phase response. Based on this CVR analysis, it has been possible to predict, in a time frame of tens of minutes, whether or not a subsequent neuronal death would occur. DHM results have been validated with trypan blue exclusion, a standard method for the determination of cell viability, which similarly to propidium iodide or the release of lactate dehydrogenase rely on the loss of membrane integrity occurring during late necrosis, after several hours.

Thus, irreversible phase responses corresponding to a CVR alteration, can be considered as an early marker of the neuronal death mediated by glutamate. In contrast, reversible phase responses corresponding to an efficient CVR are not associated with a subsequent neuronal death.

The quantitative aspects of the DHM phase signal and its high sensitivity allow to easily define quantitative criteria for discriminating healthy cells from non-viable ones. In this context, DHM may provide several advantages for cell death detection compared to standard staining techniques. Typically, as a label-free technique, it does not require any solution change or insertion of dye. Furthermore, it enables various post-processing possibilities, such as numerical optimization of imaging conditions or digital focusing, thus providing efficient conditions for high throughput screening. Finally, energy levels required for DHM can be brought typically below 0.1 W/cm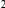, with exposure times below milliseconds, thus preventing any photo-damage even during long experiments.

## Materials and Methods

### Ethics statement

This study was carried out in compliance with the Public Health Service (PHS) Policy on Humane Care and Use of Laboratory Animals (Animal Welfare Assurance no A5692-01). Experimental procedures were approved by the Cantonal Veterinary Authorities (Vaud, Switzerland). Practically, we do not hold a permit number specifically for this study, a level 1 (the lowest) intervention. Level 1 intervention is performed as a standard procedure within the Lemanic Animal Facility Network (RESAL), the performance of which is authorized and monitored by the Cantonal Veterinary Authorities and is not subjected to the granting of a permit.

### Cell cultures

The study was performed in primary cultures of mouse cortical neurons, obtained according to the method described by Brewer et al. [35] Briefly, cortices are removed under a dissecting microscope from brains and collected in a small Petri dish in PBS-glucose. A single-cell suspension is obtained by gentle pummelling with a fire-polished Pasteur pipette in Neurobasal medium supplemented with B27 and GlutaMAX (Invitrogen). Cells are then plated at an average density of 15000 cells/cm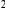 in supplemented Neurobasal medium on poly-ornithine coated glass coverslips. After 3–4 hours, coverslips are transferred to dishes containing glial cell monolayers in supplemented Neurobasal medium. Neurons are maintained at 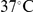 in a humidified atmosphere of 95% air / 5% 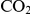 and are used after 21–35 days *in vitro*.

Prior to experiments, coverslips are mounted on a perfusion chamber used to apply the different solutions to the cells, which are immersed in a HEPES-buffered standard physiological perfusion medium containing (in mM): 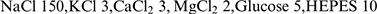, adjusted to a pH of 7.3 with NaOH. All experiments are performed at room temperature.

### Perfusion solutions

The cell viability is tested with trypan blue 0.4% reagent, diluted (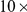) in water with 0.85% NaCl (Lonza). The dye relies on probing the cell membrane integrity, which becomes permeable to the colored compound after cell death mechanisms have been activated [36], [37]. After reagent wash-out, the cell nuclei of non-viable cells are stained in blue, as the dye fixes on DNA.

Cells are immersed during 3 minutes in a physiological medium with a 1∶10 dilution of the reagent. This low concentration and short exposure time is chosen in order to avoid staining healthy cells, as a too long exposure to trypan blue can lead to cytotoxic effects and false positives by staining viable cells [27], [28].

Excitotoxic effects are studied, by applying to the cells L-glutamate (shortly referred to as glutamate throughout the article) pulses of durations ranging typically from 1 to 2 minutes at different concentrations. Glutamate concentrations range between 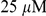 and 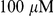, depending on the type of experiment. In each solution, 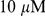 of glycine has been added, to ensure the activation of NMDA receptors by glutamate.

### Measurement setup

**Figure 7:**
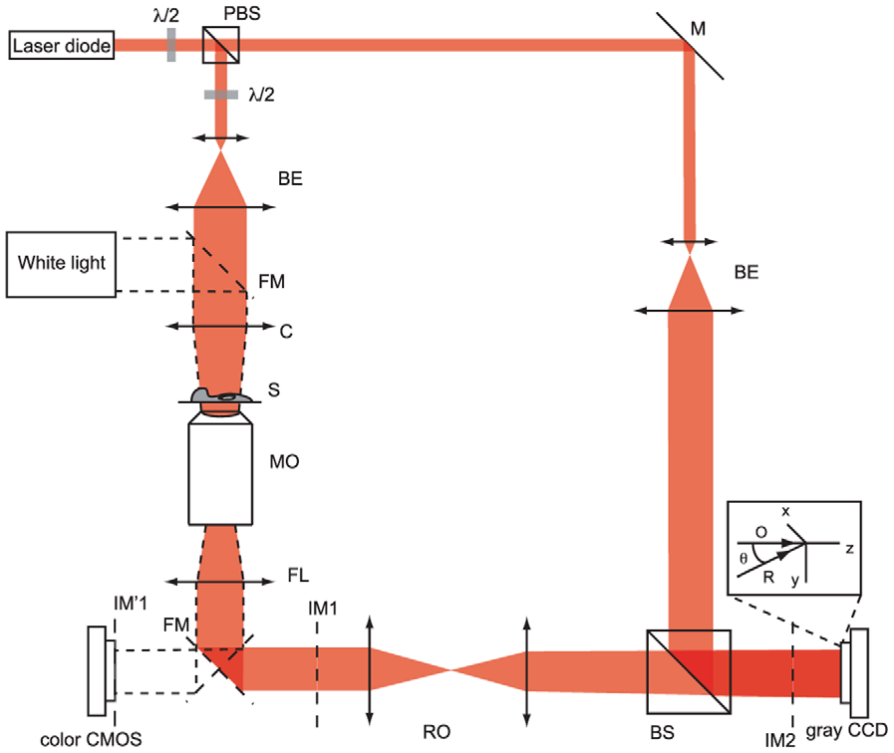
Optical setup employed for digital holography measurements. Sketch of the optical arrangement. 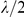: half-wavelength plate, (P)BS: (Polarizing) beam splitter, M: Mirror, FM: Flip mirror, BE: Beam expander, C: Condenser lens, MO: Microscope objective, FL: Field lens, RO: Relay optics, IM: Image plane, S: specimen, O, R: Propagation vectors of respectively object and reference waves, 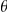: Off-axis angle.

The measurements are performed on a standard transmission DHM setup [12] enabling the monitoring of cells in time through phase measurement. A schematic representation of the optical arrangement is shown in Fig. 7, where the light emitted by a laser diode (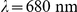), is split into two beams to generate the interference. The object beam illuminates the specimen 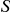, and the scattered light is collected by a 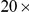 microscope objective (MO) having a numerical aperture of 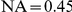 in air (Zeiss). In order to let the space for beam recombination, the image at plane IM1 is imaged through a relay optics (RO) near the CCD camera (8-bit Basler, pixel size 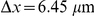) which records in the Fresnel zone the interference of the object (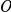) and reference (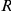) waves, having an off-axis angle 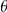 between their propagation vectors at the camera plane. The relative intensity between the two interfering beams can be adjusted through polarizing optics. Phase images are then reconstructed from holograms with standard methods for off-axis holography within 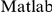
® environment as described in [16], [38]. Holograms are taken at a rate of 0.1 Hz during the experiments.

Color measurement for cell viability assessment with trypan blue requires the use of an incoherent white light, different from the laser employed for DHM. A flip mirror is thus inserted after the MO, such that the intensity image can also be recorded in focus on a color CMOS camera (8-bit, Thorlabs, 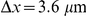), with the sample illuminated by a halogen light source. Consequently, during dye probing, no DHM measurement is performed, as both the source and camera for color measurements are different.

Image merging between the two measurement techniques could be done through calibration of the system by imaging an object with a well-known shape. Due to the RO employed for DHM measurements and to the different pixel sizes of both cameras, a scaling and a translation have to be performed in order to obtain proper merged images.

## Supporting Information

Video S1
**Time course monitored with digital holography.** Media file showing the time-course of quantitative phase images presenting neurons under an excitotoxic glutamate pulse (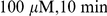), where the well-known cell swelling can be identified through the increase in size and intracellular dilution. On a second stage, cell blebbing and then nucleus condensation occur, due to cell death mechanisms triggering. Gray images in the movie correspond to trypan blue assessment periods, where no phase measurement is performed.(MPG)
